# Screening of triploid with low-coverage whole-genome sequencing by a single-nucleotide polymorphism-based test in miscarriage tissue

**DOI:** 10.1007/s10815-019-01588-6

**Published:** 2019-11-13

**Authors:** Qian Geng, Xiaoli Cui, Yaqi Zhang, Lijuan Zhang, Cai Zhang, Kai Wang, Jianguo Chen, Qingyan Zhu, Jiansheng Xie, Zhiyong Xu, Yang Liu, MengMeng Zhang, Lijie Ding, Wenyong Zhang, Chuanchun Yang

**Affiliations:** 1 Shenzhen Maternity and Child Healthcare Hospital, Shenzhen, China; 2 CheerLand Precision Biomed Co.,Ltd, Shenzhen, Guangdong China; 3 Southern University of Science and Technology, Shenzhen, China; 4 Southern University of Science and Technology-CheerLand Institute of Precision Medicine, Shenzhen, Guangdong Sheng China

**Keywords:** Single-nucleotide polymorphism, Heterozygous mutation, Quadratic curve, *Z* test, Triploid, Uniparental disomy

## Abstract

**Purpose:**

To establish a single-nucleotide polymorphism-based analysis (SBA) method to identify triploidy in the miscarriage tissue by using low-coverage whole-genome sequencing (LC-WGS).

**Methods:**

The method was established by fitting a quadratic curve model by counting the distribution of three heterozygous mutation content intervals. The triploid test result was mainly determined by the opening direction and the axis of symmetry of the quadratic curve, and *Z* test between the same batch samples was also used for auxiliary judgment.

**Results:**

Two hundred thirteen diploid samples and 8 triploid samples were used for establishment of the analytical method and 203 unknown samples were used for blind testing. In the blind testing, we found 2 cases positive for triploidy. After chromosome microarray analysis (CMA) and mass spectrometry verification, we found that both samples were true positives. We randomly selected 5 samples from the negative samples for mass spectrometry verification, and the results showed that these samples were all true negatives.

**Conclusions:**

Our method achieved accurate detection of triploidy in the miscarriage tissue and has the potential to detect more chromosomal abnormality types such as uniparental disomy (UPD) using a single LC-WGS approach.

## Introduction

Chromosomal abnormalities generally refer to structural or copy number variations that can lead to a number of serious health outcomes such as infertility [1, 2], recurrent miscarriage [3], birth defect, and cancer [4]. In all clinically recognized pregnancies, approximately 10–15% result in abortions, and most of abortions occur in the first trimester. About 50% of early pregnancy abortions are caused by chromosomal abnormalities [5], most of which (86%) are due to abnormal chromosome numbers, including trisomy, monosomy, and polyploidy. Triploidy (69, XXX; 69, XXY; 69, XYY), which refers to an extra set of chromosomes present in each cell, is one of the most common chromosomal abnormalities [6–8]. About 1–2% of pregnancies and 10% of early abortions are caused by triploidy [5, 8–10]. Most triploid fetuses are unable to survive pregnancy and undergo spontaneous abortion between the 7th and 17th weeks of pregnancy [6, 8, 11, 12]. Live birth of triploid babies is very rare and they usually die early after birth with only a few reported cases of unusually long survival [13].

Considering that the phenomenon of triploidy is quite common in pregnancy and has serious consequences, it is of clinical significance to develop a technique that can detect this abnormality accurately. There are many techniques available for detecting chromosomal abnormalities, such as karyotyping, fluorescence in situ hybridization (FISH), and chromosome microarray analysis (CMA). FISH can confirm the locations of chromosomal abnormalities identified by CMA, targeted next-generation sequencing (NGS), and WGS, but fails to detect de novo chromosomal abnormalities. The CMA technology has been widely used to study abortion causes [6, 14], but is limited by failure to detect certain chromosome rearrangements, such as balanced translocations and inversions. Recently, using paired reads that span breakpoints, targeted NGS and WGS technology has been applied to detect chromosomal abnormalities [15, 16]. As sequencing cost continues to decline, WGS has been widely used to investigate the cause of miscarriage. In general, greater the depth of WGS, more accurate the analysis will be. However, as the depth of sequencing increases, so does the cost. It is a balance of accuracy and cost that will determine the depth of sequencing employed. Given the technologic advances, it is of great value to develop a comprehensive detection method for chromosomal abnormalities including CNV, aneuploidy, triploidy, and UPD using a single NGS method such as LC-WGS.

## Materials and methods

### Study design

#### Phase 1—Establishment of methodology

First, genomic DNA isolated from 8 triploid miscarried products of conception (POC) and genomic DNA from 213 diploid individuals were sequenced using LC-WGS. The establishment of methodology was developed in the following steps. In short, we determined the triploidy status according to the proportion of mutant reads in each heterozygous SNP. First of all, heterozygous sites were selected for each sample. We defined the ratio of the number of mutated reads to the number of all reads at the site as mutation ratio (MR). Since there are two copies of each autosomal chromosome in diploid cells, MR should be close to 1/2 in diploid cells. In contrast, for triploid cells, 1/3 and 2/3 MR for heterozygous SNPs are expected. However, due to sequencing variabilities, the MR of each SNP site is not exactly 1/3 or 2/3, so we defined “1/3 interval” as [0.28, 0.38], “2/3 interval” as [0.62, 0.72], and “1/2 interval” as [0.45, 0.55]. Next, the number of SNPs belonging to three intervals was respectively counted as SNs. We set up a coordinate system with MR as abscissa and SNs as ordinate. For each sample, we got three data points: (1/3, y1), (1/2, y2), (2/3, y3). The parabolic equation *y* = *ax*^2^ + *bx* + *c* was fitted to these data points. When the sample is triploid, the opening direction of parabola is upward, *a* > 0; when the sample is diploid, the opening direction of parabola is downward, *a* < 0. The central axis of the parabola has an abscissa of –*b*/2*a* and the value should be within [0.45, 0.55].

#### Phase 2—Validation of SBA analysis

A blinded test was performed in order to evaluate the feasibility of the SBA method in differentiating diploids and triploids at low sequencing depth. To avoid the absence of triploidy in the blinded samples, we mixed a triploid sample into 202 samples of unknown type. These 203 samples were blindly tested using the SBA method. The triploids detected by SBA were subsequently verified by mass spectrometry genotyping method and CMA. In addition, 5 negative samples from blinded samples were selected randomly to be verified by mass spectrometry analysis.

#### Patients and sample collection

All samples of abortion tissue involved in this study were obtained in SZMHH from January 1, through August 1, 2013. The tissue was frozen by liquid nitrogen treatment and DNA was isolated and stored in the − 80 °C refrigerator immediately after it was isolated. All samples were analyzed at Beijing Cheerland Medical Laboratory Co., Ltd., which is certified by the National Health Commission of the People’s Republic of China.

#### Library construction and sequencing

The aborted tissue was washed with phosphate-buffered saline (PBS) after being thawed. Genomic DNA was extracted by QIAamp DNA mini kit (Qiagen, Hilden, Germany). Quantity and purity of gDNA were assessed by Qubit 3.0 fluorometer (Invitrogen, Carlsbad, CA, USA) and NanoDrop-One (Thermo Scientific, Wilmington, DE, USA). One microgram of gDNA was fragmented to the size range of 200–500 bp by M220 Focused-ultrasonicator (Covaris, UK), and 100–300-bp DNA fragments were selected using AMPure XP beads (Agencourt, CA, USA). Then, the selected DNA fragments were repaired and modified at 3′ end. The dTTP tail junction sequence was ligated to the end of DNA fragment, and the DNA fragment was amplified for 8 cycles and subjected to a single-strand cyclization process. The PCR product was denatured with a specific molecule is then ligated by DNA ligase. The remaining linear molecules were digested by exonuclease, and finally a single-stranded loop DNA library was obtained. The generated sequencing library was quality control by Agilent 2100 bioanalyzer (Agilent, CA, USA) and Qubit 3.0 (Invitrogen). All samples were subjected to 50-bp pair end sequencing using the BGISEQ-500 sequencing platform.

### Data analysis

#### Phase A—Mutation detection and quality control

The raw paired reads for each sample were approximately 60 M. All reads were inversely adjusted, and the inverted reads were merged with the original data to form a new data set for triploid analysis. First, data filtering was performed using SOAPnuke (version 1.5.0). After filtering for clean reads, the data were mapped to the reference genome sequence (hg19) using BWA (0.7.12-r1039). Variation detection and filtration were performed by GATK HaplotypeCaller. Then we combined the filtered INDEL and SNP. Finally, data size and average depth of each chromosome were compared for statistical and quality control.

#### Phase B—SNP filtering

We screened the SNPs for each sample. All SNPs that were used to calculate the parabola must satisfy both of the following conditions. First, it must exist in the dbSNP database (version 138), and the frequency in the 1000 Genomes database (phrase 3 version) needs to be more than 5%. At the same time, the number of reads must be more than 10 after the inversion. Through the above two steps of screening, we obtained available SNPs for triploidy analysis.

## Results

### Establishment of analysis method

For phase 1 of the study, a total of 213 diploid and 8 known triploid samples, whose karyotypes had been confirmed by CMA, were sequenced. After SBA analysis, the results were highly consistent with CMA results (Table 1) and only one false positive case was seen due to DNA degradation and potential maternal cell contamination. Overall, our method achieved 100% sensitivity and 99.53% specificity using this sample set. Furthermore, the calculated parameters for triploid and diploid samples were significantly different and were sufficient to correctly identify triploidy (Table 2).

**Table 1:** Parabolic data for triploid and diploid

Items	Positive		Negative	
Test results	True positive(*a*)	8	False positive(*b*)	1
	False negative(*c*)	0	True negative(*d*)	212
Sensitivity	*a*/(*a* + *c*) × 100%		100(%)	
Specificity	*d*/(*b* + *d*) × 100%		99.53(%)	
Accuracy	(*a* + *d*)/(*a* + *b* + *c* + *d*) × 100%		99.55(%)

**Table 2:** Parabolic data for triploid and partial diploid

Sample	(1/3 + 2/3)/(1/2)	*a*	− *b*/2*a*	Mean	SD	*Z*-score	Karyotype
HMC_1	2.29	1.61	0.55	1.77	0.09	5.83	68,XX
HMC_2	2.27	1.47	0.54	1.77	0.09	5.52	69,XXY
HMC_3	2.24	1.35	0.55	1.77	0.09	5.25	69,XXX
HMC_4	2.26	1.42	0.54	1.77	0.09	5.41	69,XXX
HMC_5	2.20	1.14	0.53	1.77	0.09	4.81	68,XXY,-21
HMC_6	2.21	1.16	0.54	1.77	0.09	4.86	69 XYY
HMC_7	2.24	1.32	0.55	1.77	0.09	5.20	69,XXY
HMC_8	2.16	0.91	0.56	1.77	0.09	4.34	69,XXX
HMC_9	2.20	1.13	0.55	1.77	0.09	4.80	46,XY
HMC_9*	1.79	− 1.35	0.53	1.77	0.09	0.23	46,XY
Case_1	1.80	− 1.25	0.48	1.77	0.09	0.38	46,XX
Case_2	1.76	− 1.59	0.49	1.77	0.09	− 0.14	46,XX
Case_3	1.86	− 0.9	0.48	1.77	0.09	0.96	46,XY
Case_4	1.90	− 0.64	0.47	1.77	0.09	1.41	46,XY
Case_5	1.83	− 1.05	0.49	1.77	0.09	0.72	46,XX

*The HMC_9 sample was tested twice, and the sample DNA of first test was likely to be contaminated or degraded

**Fig. 1:**
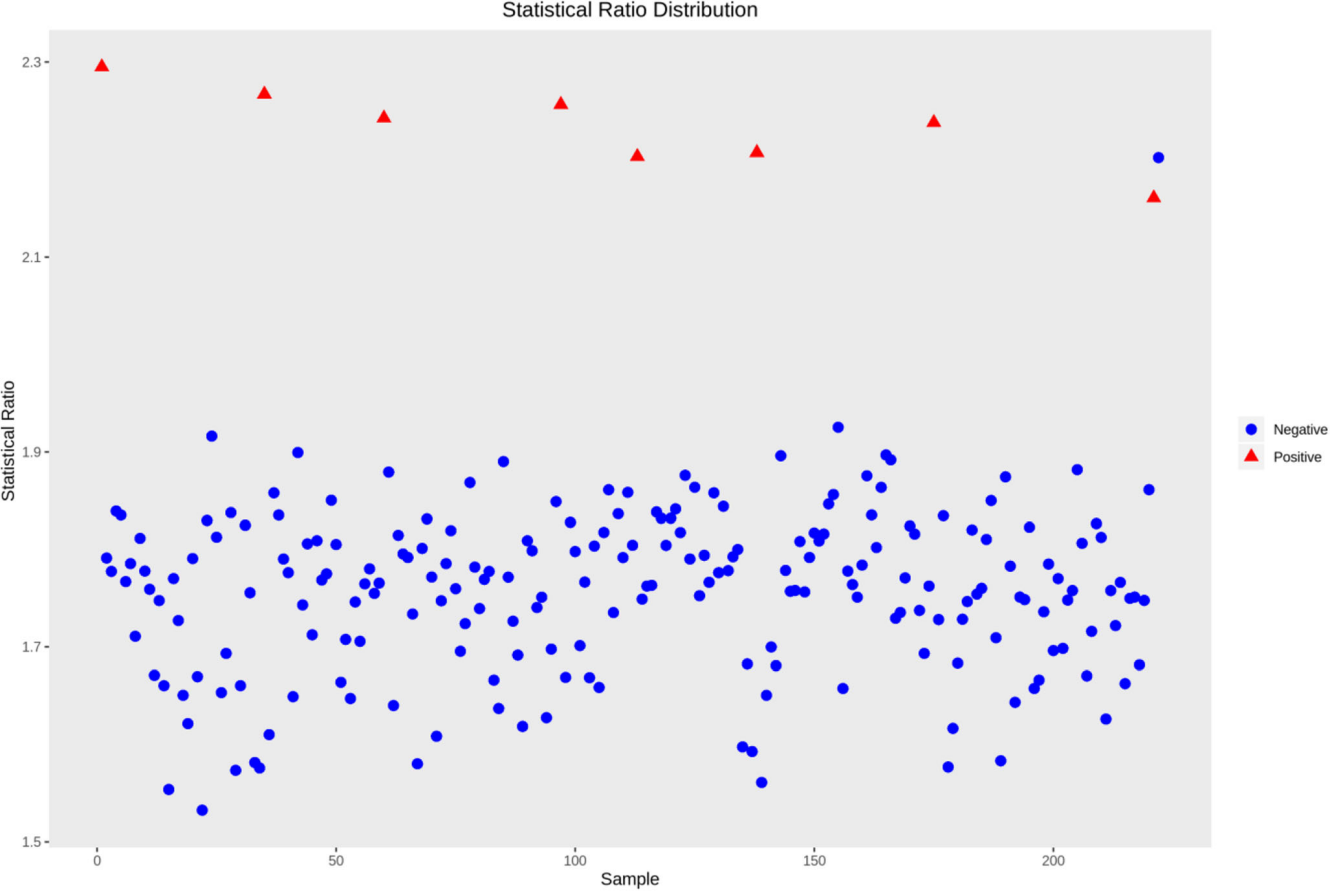
The ratio between the number of sites with a MR value of 1/3 or 2/3 and the number of sites with a MR value of 1/2. From this ratio, triploid and diploid are clearly divided into two categories. Red triangles represent positive samples and blue circles represent negative samples. The false positive sample in the upper right is contaminated HMC_9 sample

We conducted clustering analysis and found that triploids and diploids were completely separated with a calculated value 2 as the cutoff (Fig. 1). Triploids were clearly clustered above 2, and diploid was clearly clustered below 2.

### Performance of the analysis method on 203 unknown samples

**Fig. 2:**
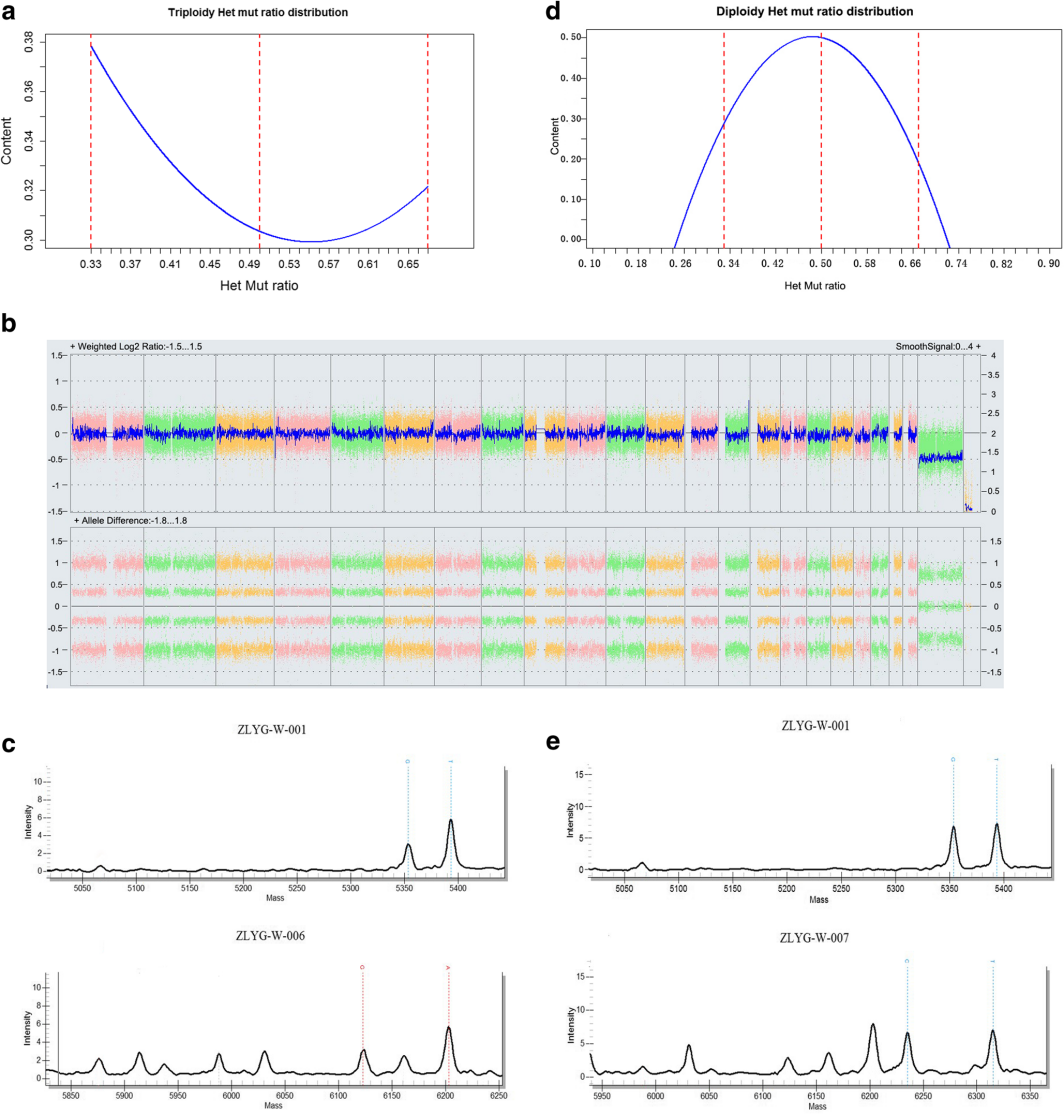
Blind samples were analyzed by SBA, CMA, and mass spectrometry. **a** Results of positive samples (HCM011, 68,XX) obtained by SBA analysis showed that the opening direction of the parabola was upward and the symmetry axis was within [0.45, 0.55]. **b** Results obtained by CMA testing are considered to be triploid in terms of mutation frequency. **c** Points from dbSNP database with higher mutation frequency in the population were selected for mass spectrometry verification. The results showed high and low peaks with a peak ratio close to 1:2, consistent with triploidy. **d** The result of negative sample (HMC067) obtained by SBA analysis shows that the opening direction of the parabola is upward and the symmetry axis is within [0.45, 0.55]. **e** Five cases were randomly selected from the negative samples for mass spectrometry verification. In the mass spectrometry figures of one representative sample (HMC067), equal peaks of heterozygous sites were found, and the ratio of peak height was close to 1:1, consistent with diploidy

To test the reliability of this method, 203 samples were blindly detected using the SBA method. To prevent the absence of triploids in these samples, we mixed a triploid sample into 202 unknown samples. Form these 203 samples, 2 tripoids and 201 diploids were detected (Fig. 2a, d). CMA and mass spectrometry genotyping assay were performed on the two triploid samples to verify our findings, and the results showed that the two samples were true positive for triploidy (Fig. 2b, c). In addition, we randomly selected 5 out of 201 diploid samples for mass spectrometry assay validation. For the SNP sites with high-frequency mutation, 26 PCR primers were designed and the amplification products were verified by mass spectrometry (Fig. 2e). It can be seen from the results of mass spectra that the peaks of the 5 negative samples were all single peaks or equal peaks, so that these 5 samples were true negative for tripoidy.

### Detection of UPD using SBA method

**Fig. 3:**
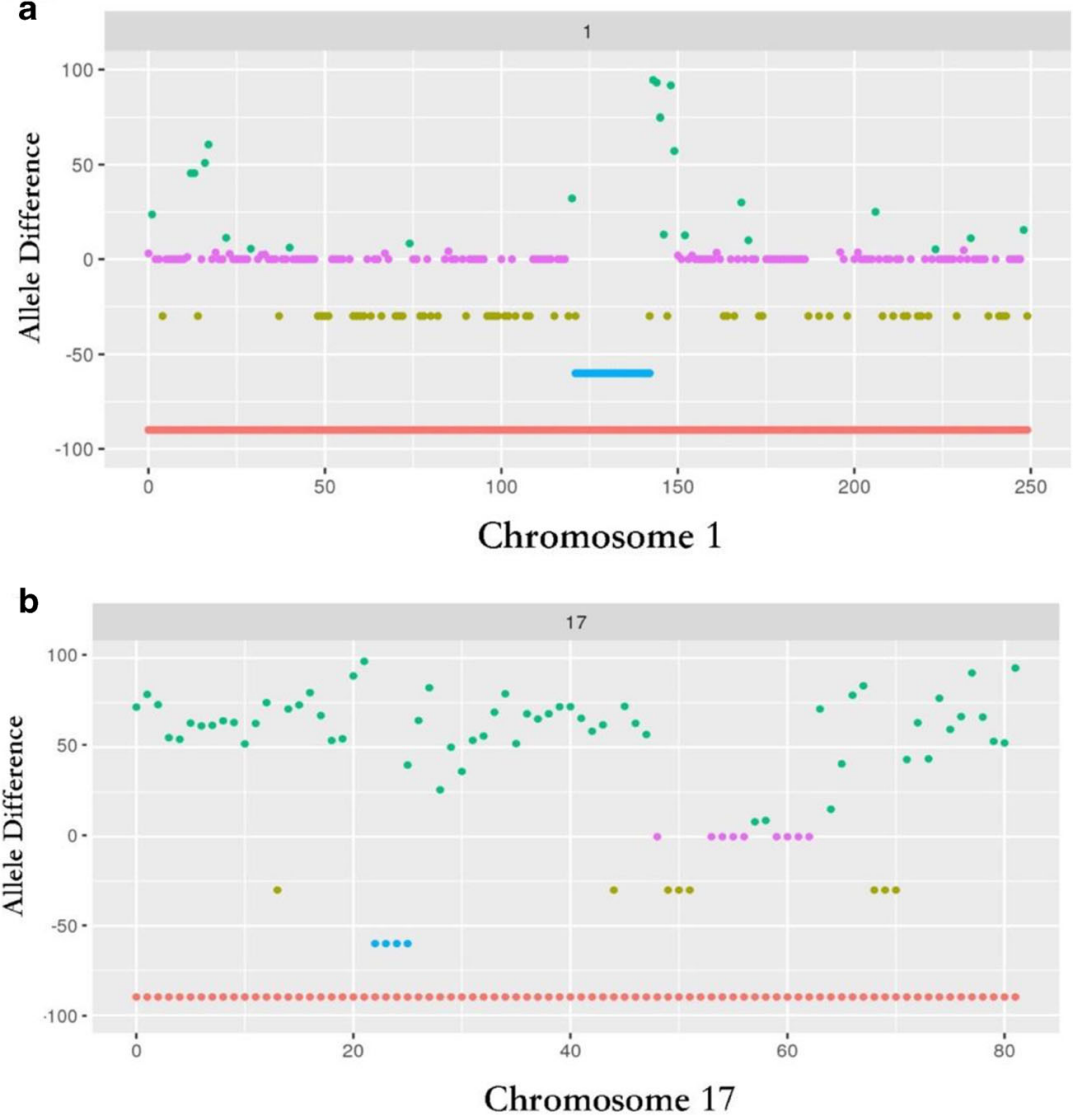
The *x*-coordinate represents the section of the chromosome. The vertical coordinate represents the percentage (frequency) of heterozygous SNPs. There are five colors of dots in the figure, and each representing a 1-Mb section. The green points indicate that the hybrid SNP frequency in the 1-Mb segment is greater than 5%, the pink points indicate that the hybrid SNP frequency is between 0 and 5%, and the yellow points indicate the section of poor data quality, which is not suitable for subsequent analysis. The blue interval represents the Gap region on the chromosome. The orange stripe is the length of the entire chromosome. **a** Uniparental disomy on chromosome 1 (sample HCM056, 46, XX). **b** Segmental uniparental disomy of chromosome 17, 17q21.33–q23.3 (sample HCM056, 46, XX)

UPD refers to the situation where two homologous chromosomes or segments of homologous chromosomes coming from the same parental source [17]. UPD can be either congenital or acquired, with the latter case occurring usually in tumors [18]. Most of the UPDs are not inherited from their parents but are associated with abnormalities in meiosis, fertilization, and mitosis [19, 20]. In this study, we found a sample without chromosomal deletions, duplications, translocation, or inversion. SBA analysis was performed for these samples. Frequency distribution of heterozygous SNPs in each chromosomal segment was counted. The heterozygous SNP frequencies (HSFs) in entire chromosome 1 were between 0 and 5%, indicating a chromosome 1 UPD (Fig. 3a). We also found that HSFs in region 17q21.33–q23.3 were mostly between 0 and 5%, indicating that segmental UPD involving 17q21.33–q23.3 (Fig. 3b).

## Discussion

Several technologies such as cytogenetics, CMA, FISH, and WGS can be used to investigate chromosomal abnormalities. Cytogenetic analysis of cultured chorionic or fetal tissues is still regarded as gold standard for chromosomal ploidy analysis. However, this technology has shortcomings such as difficulties in tissue culture, contamination from maternal cells, and time inefficiency. Some new technologies have emerged, including array comparative genomic hybridization (aCGH), and multiplex ligation-dependent probe amplification (MLPA) [21]. Microarray-based method can detect millions of genomic loci simultaneously with high resolution and is particularly suitable for detection of micro duplications/deletions. NGS-based CNV-Seq is mainly used to analyze chromosome aneuploidy, including microdeletions/duplications and trisomy, but still faces challenges in triploidy detection. In this study, we developed a tripoidy detection method by analyzing the frequency distribution of heterozygous SNPs in the sample by taking advantage of LC-WGS technology. Our method can detect triploidy with high accuracy. Moreover, we envision that our method’s utility is not limited to the detection of triploidy and also has the potential for the detection of other chromosomal abnormalities such as UPD.

Although our method can detect triploidy in majority of tested cases, there are situations that will negatively affect the accuracy of triploidy detection. For example, if fetal samples contain significant maternal contamination or are positive for somatic mosaicism, the accuracy of analysis will decrease. When maternal blood is mixed into the triploid sample, the tripoidy signal will be reduced, leading to potential false negative results. Therefore, maternal cell contamination should be avoided as much as possible. On the data analysis level, we can use the proportion of X chromosome to help us rule out potential maternal contamination. If the proportion of X chromosome is abnormally high, we should consider the possibility of maternal cell contamination. Somatic mosaicism also posts significant analytic challenge using our LC-WGS method.

**Fig. 4:**
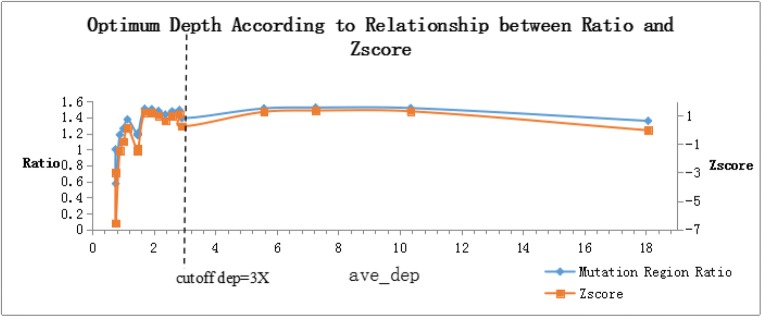
Thresholds for the amount of data available for analysis. The *X*-axis represents the sequencing depth. The *Y*-axis on the right is the ratio of the number which the MR value is in the “1/3 interval” and the “2/3 interval” to the number which the MR value is in the “1/2 interval”. Since the sample shown in the figure is a non-triploid sample, the smaller the ratio, the more accurate the result will be. The blue line represents the ratio curve of the non-triploid samples, and the red line represents the *Z*-score. It can be seen from the figure that when the depth is 3×, the curve tends to be stable and the overall trend of *Y* value decreases

In order to increase analysis accuracy, one option is to increase sequencing depth. However, increased sequencing depth comes with higher cost. To balance accuracy and cost, we investigated the desirable sequencing depth for tripoidy detection in non-mosaic samples. We found that sequencing depth of 3 is required for successful tripoidy analysis (Fig. 4).

In summary, we successfully developed a LC-WGS-based SBA method that allows accurate detection of tripoidy in 203 blinded POC samples. We also provided proof of concept that this method could be used for whole chromosomal or segmental chromosomal UPD detection. Combining low sequencing depth and WGS, this method offers high detection accuracy, wide utility, and reasonable cost. As the sequencing cost of WGS continues to decrease, we foresee that our method will provide more values in the analysis of chromosomal abnormalities as well as mutation detection to aid the study to identify causes of infertility, abortion, and genetic diseases.
